# An optimized prediction framework to assess the functional impact of pharmacogenetic variants

**DOI:** 10.1038/s41397-018-0044-2

**Published:** 2018-09-12

**Authors:** Yitian Zhou, Souren Mkrtchian, Masaki Kumondai, Masahiro Hiratsuka, Volker M. Lauschke

**Affiliations:** 1 Department of Physiology and Pharmacology, Section of Pharmacogenetics, Karolinska Institutet, SE-171 77 Stockholm, Sweden; 2 Laboratory of Pharmacotherapy of Life-Style Related Diseases, Graduate School of Pharmaceutical Sciences, Tohoku University, Sendai, Japan

**Keywords:** Pharmacogenomics, Personalized medicine

## Abstract

Prediction of phenotypic consequences of mutations constitutes an important aspect of precision medicine. Current computational tools mostly rely on evolutionary conservation and have been calibrated on variants associated with disease, which poses conceptual problems for assessment of variants in poorly conserved pharmacogenes. Here, we evaluated the performance of 18 current functionality prediction methods leveraging experimental high-quality activity data from 337 variants in genes involved in drug metabolism and transport and found that these models only achieved probabilities of 0.1–50.6% to make informed conclusions. We therefore developed a functionality prediction framework optimized for pharmacogenetic assessments that significantly outperformed current algorithms. Our model achieved 93% for both sensitivity and specificity for both loss-of-function and functionally neutral variants, and we confirmed its superior performance using cross validation analyses. This novel model holds promise to improve the translation of personal genetic information into biological conclusions and pharmacogenetic recommendations, thereby facilitating the implementation of Next-Generation Sequencing data into clinical diagnostics.

## Introduction

In the last decades, rapid progress in sequencing technologies has allowed the deciphering of genomic information on an unprecedented scale. While the initial sequencing of the human genome in the frame of the Human Genome Project cost 2.7 billion USD and took 14 years to complete, costs and times declined to around 1200 USD and 1.5 days for a whole-genome sequence with 30× coverage in 2015 [1] and technology to enable the 100 USD genome has already been announced [2]. As outcomes of these technological advancements, the vast extent of genomic information has propelled medicine by providing information about disease susceptibility, e.g. in cancer [3, 4], type 2 diabetes mellitus [5] or schizophrenia [6], by identifying genes that underlie monogenic disorders [7, 8] and by facilitating the discovery of novel therapeutic targets, particularly in oncology [9].

However, despite these successes of human genomics on a population scale, the translation of personal genomic data into clinically actionable information remains difficult. Each individual harbors on average 23,000–25,000 genetic variants in exons, including 10,000–12,000 variants resulting in amino acid exchanges and around 100 variants resulting in stop-gain mutations, frameshifts or differential splice sites, the vast majority of which are rare with minor allele frequencies (MAF) < 1% [10]. Genes with importance for drug absorption, distribution, metabolism and excretion (ADME) are highly variable [11–13] and such genetic variability has been estimated to account for around 20–30% of the inter-individual differences in drug response [14]. However, while on average around 100 genetic variants are detected across ADME genes in each individual, the overwhelming majority has not been experimentally characterized, which poses a significant challenge for the clinical interpretation of genetic variability and impairs the translation of genomic data into actionable advice [15, 16].

As systematic experimental analyses in relevant expression systems are hitherto not feasible for these vast numbers of variants, computational methods have been proposed for predicting the functional relevance of identified genetic mutations. In recent years, dozens of algorithms have been presented that aim to distinguish deleterious from neutral variants. These algorithms use a variety of features, such as secondary structure, functional sites, protein stability or sequence conservation, and are mostly based on machine learning techniques, such as support vector machines, artificial neural networks or naïve Bayes classifiers [17–19]. Importantly, computational methods are generally trained on sets of variants with high evolutionary constraints implicated in disease. However, as many ADME genes are generally only poorly conserved, we hypothesize that specialized pharmacogenetic prediction models are needed that have been calibrated on appropriate ADME data sets.

In this study, we used experimental activity data from 337 variants distributed across 43 ADME genes to evaluate current functionality prediction methods and found that standard algorithms are only relatively poor predictors of the functional impact of ADME gene mutations. We thus developed a novel computational functionality prediction model optimized for pharmacogenetic assessments, which substantially outperformed standard algorithms, correctly flagging 93% of experimental loss-of-function (LOF) variants as deleterious and 93% of variants without functional impact as neutral. Thus, the ADME-optimized prediction framework significantly improves in silico functionality assessment of pharmacogenetic variants, thereby facilitating the translation of uncharacterized variants into pharmacogenetic recommendations and providing a further step towards the leveraging of Next-Generation Sequencing data for the personalization of pharmacological treatment.

## Methods

### In vitro functionality data

We obtained experimental functionality data for 337 single variant alleles from the 43 ADME gene (see Supplementary Table 1 for references). The common variants rs3758581 (CYP2C19 I331V), rs16947 (CYP2D6 R296C) and rs1135840 (CYP2D6 S486T) were considered as neutral. An overview of all analyzed variants, the substrates and expression systems used for characterization and the in silico predictions by all tested algorithms is provided in Supplementary Table 2. Wherever necessary, variant coordinates were translated to a uniform reference genome version. Mutations for which no score could be retrieved by any prediction method were excluded. Variants were considered to have a deleterious impact if they reduced their intrinsic clearance more than 2-fold compared to the wildtype allele (for most genes the *1, in the case of *NAT1* the *4 allele).

### Statistical definitions

True positives (TP) and false negatives (FN) are variants that have a functional impact in vitro and are predicted in silico to be deleterious or neutral, respectively. Conversely, true negatives (TN) and false positives (FP) are defined as mutations that do not affect the functionality of the gene in vitro and are predicted in silico to be neutral or deleterious, respectively. The true positive rate or sensitivity is defined as $\frac{{\sum TP}}{{\sum TP + \sum FN}}$, specificity is $\frac{{\sum TN}}{{\sum TN + \sum FP}}$ and the false positive rate is defined as $\frac{{\sum FP}}{{\sum TN + \sum FP}}$. Furthermore, the positive and negative predictive values are calculated as $\frac{{\sum TP}}{{\sum TP + \sum FP}}$ and $\frac{{\sum TN}}{{\sum TN + \sum FN}}$, respectively and the total predictive accuracy is $\frac{{\sum TP + \sum TN}}{{\sum TP + \sum TN\sum FP + \sum FN}}$.

### Computational functionality predictions

We compared the functionality assessments of 18 current in silico functionality prediction algorithms, conservation scores and ensemble scores computed using ANNOVAR:[20] SIFT [21], PolyPhen-2 [22], Likelihood ratio tests [23], MutationAssessor [24], FATHMM [25], FATHMM-MKL [26], PROVEAN [27], VEST3 [28], CADD [29], DANN [30], MetaSVM [31], MetaLR [31], GERP++ [32], SiPhy [33], PhyloP [34] (using both vertebrate and mammalian alignments) and PhastCons [35] (using both vertebrate and mammalian alignments).

### Development of ADME optimized algorithm

**Fig. 1:**
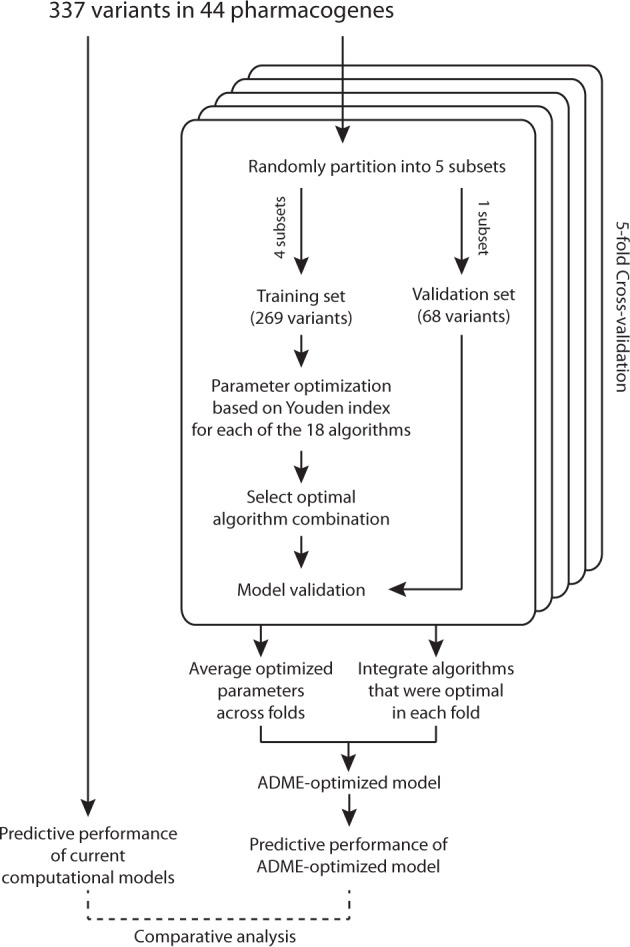
Schematic depiction of the workflow for the development of the ADME optimized prediction model

The 337 alleles were randomly partitioned into five subsets for 5-fold cross validations while assuring equal proportions of deleterious and neutral variants (Fig. 1). Thresholds for the individual algorithms were optimized on the basis of the Youden index or informedness function, which can be interpreted as the probability of an informed classification. The Youden index, defined as *I* = sensitivity + specificity – 1, was calculated for each potential threshold (increments 0.01–0.05) between the highest and lowest possible scores for each respective method. All variants *i* were classified as deleterious or neutral by each of the k threshold-optimized algorithms. If the computational prediction for var_i_ aligns with the corresponding experimental result, then score s_k,i_ = 1 otherwise s_k,i_ = 0. Subsequently, out of all possible constellations the algorithm combination was selected for the ADME-optimized model for which $\mathop {\sum}\nolimits_i {\mathop {\sum}\nolimits_l {s_{l,i}} } = max$ with l ≤ k. Importantly, the result with this model was validated for each fold using the independent validation set. Overall, optimal results for the pharmacogenetic prediction model were derived by integrating assessments of LRT, MutationAssessor, PROVEAN, VEST3 and CADD. The overall prediction score of the ADME -optimized model is defined as follows: each of the algorithms predicts whether a variant is deleterious or neutral based on its ADME-optimized threshold value (1 = deleterious and 0 = functionally neutral). The final score is derived by averaging the assessments of the individual algorithms (1 or 0). Thus, a score of 1 indicates that all algorithms predicted the variant to be deleterious, a score of 0 that all algorithms predicted the variant to be neutral and a score of e.g. 0.5 that half of the algorithms predicted the variant to be deleterious and half to be neutral. Receiver operating characteristics (ROC) analyses were performed using Prism 6 (GraphPad Software Inc.).

## Results

### Conventional computational algorithms have a low predictive accuracy when applied to pharmacogenetic variants

**Fig. 2:**
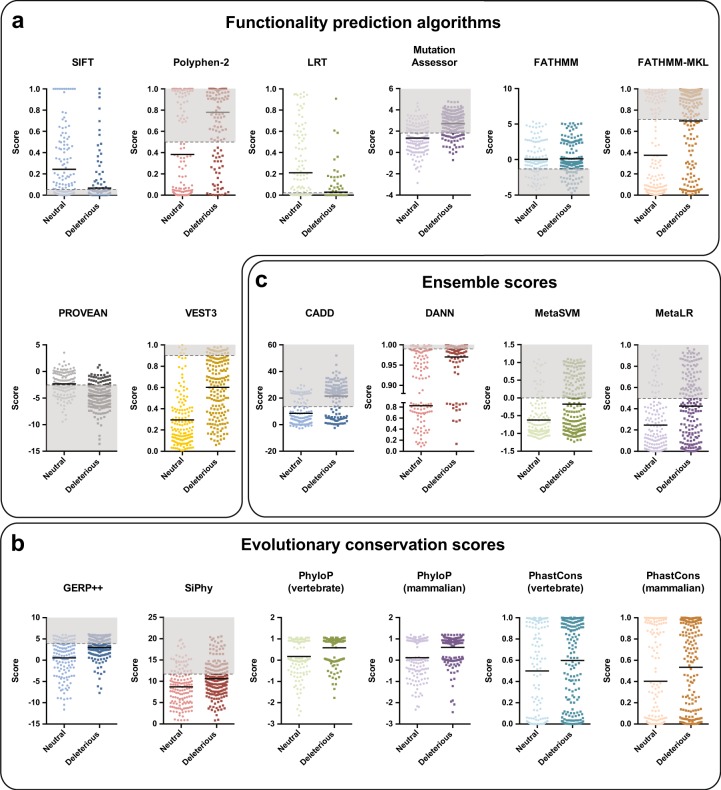
Overview of the performance of different functionality prediction methods. Variants (*n* = 337) were separated into phenotypically neutral variants (lighter shaded circles) and those that have a relevant impact on substrate metabolism (intrinsic clearance reduced >2-fold; darker shaded squares). **a**–**c** Functionality was predicted using eight common prediction algorithms (**a**), 6 evolutionary conservation scores (**b**) and 4 ensemble scores (**c**). Conventional thresholds of the respective algorithms are depicted as dashed lines and intervals of functionality scores deemed functional are shaded in light gray. The average scores of variants in the neutral and deleterious groups are indicated

We first evaluated the performance of current computational functionality assessment algorithms on pharmacogenetic variants across 43 ADME genes with low evolutionary constraints (Supplementary Table 3). To this end, we derived predictions for 337 pharmacogenetic single nucleotide variants (SNVs) with available high-quality experimental data. These variants cause alterations in the amino acid sequence of their corresponding gene product, which can either cause direct modulation of protein activity, result in changes in protein levels, for instance due to misfolding followed by degradation or entail dysregulation of protein transport. We evaluated eight commonly used functionality prediction algorithms, SIFT, PolyPhen-2, LRT, MutationAssessor, FATHMM, FATHMM-MKL, PROVEAN and VEST3 (Fig. 2a). When using the area under the ROC curve (AUC_ROC_) as measure for model quality, VEST3, MutationAssessor and PolyPhen-2 exhibited the best performance with AUC_ROC_ values of 0.8, 0.78 and 0.77, respectively, whereas FATHMM performed worst (AUC_ROC_ = 0.51; Table 1).

**Table 1:** Comparison of the predictive performance of functionality prediction tools on pathogenic and pharmacogenetic data sets

Algorithm	Category	Performance on disease-associated data set (AUC_ROC_)	Performance on pharmacogenetic data set (AUC_ROC_)
SIFT	Functionality prediction algorithms	0.76–0.88	0.74
PolyPhen-2		0.79–0.88	0.77
LRT		0.67–0.72	0.75
MutationAssessor		0.8–0.83	0.78
FATHMM		0.87–0.91	0.51
FATHMM-MKL		0.91	0.73
PROVEAN		0.85	0.76
VEST3		0.91	0.8
GERP++	Evolutionary conservation scores	0.67–0.78	0.67
SiPhy		0.69–0.81	0.63
PhyloP (vertebrate)		0.67–0.83	0.64
PhyloP (mammalian)			0.64
PhastCons (vertebrate)		0.67–0.83	0.58
PhastCons (mammalian)			0.61
CADD	Ensemble scores	0.93	0.81
DANN		0.95	0.75
MetaSVM		0.88–0.89	0.68
MetaLR		0.92–0.94	0.68

Performance measures on disease-associated data sets were obtained from refs. [26–31]

Next, we tested the performance of four models, GERP++, SiPhy, PhyloP and PhastCons using different phylogenetic models (using 7 vertebrates or 20 mammals), resulting in a total of six sores that use evolutionary conservation based on sequence alignments as a measure for functional importance (Fig. 2b). Overall, the predictive power of evolutionary conservation scores (AUC_ROC_ = 0.58–0.67) was substantially lower than that of functionality prediction algorithms which base their assessment also on additional features, such as homology alignments or structure-based features (AUC_ROC_ = 0.51–0.8; Table 1). These findings suggest that evolutionary conservation alone seems to be a poor indicator of functional impact in poorly conserved loci, such as ADME genes.

We furthermore analyzed the ensemble scores CADD, DANN, MetaSVM and MetaLR that integrate assessments from multiple orthologous methods (Fig. 2c). CADD and DANN performed substantially better than MetaSVM and MetaLR on our data set with the former showing the best predictive performance of all models analyzed (AUC_ROC_ = 0.81; Table 1). Importantly, the predictive power of most algorithms on our ADME variant cohort was substantially lower compared to data sets based on pathogenicity-associated variants (Table 1), emphasizing the shortcomings of model parameterization based on genome-wide analyses for pharmacogenetic functionality predictions.

### Optimization of pharmacogenetic functionality predictions

To improve the predictive power of pharmacogenetic functionality predictions, we structured the problem into two tasks: first, we optimized the classification thresholds of the individual algorithms and, in a second step, we selected the optimal combination of model components.

**Fig. 3:**
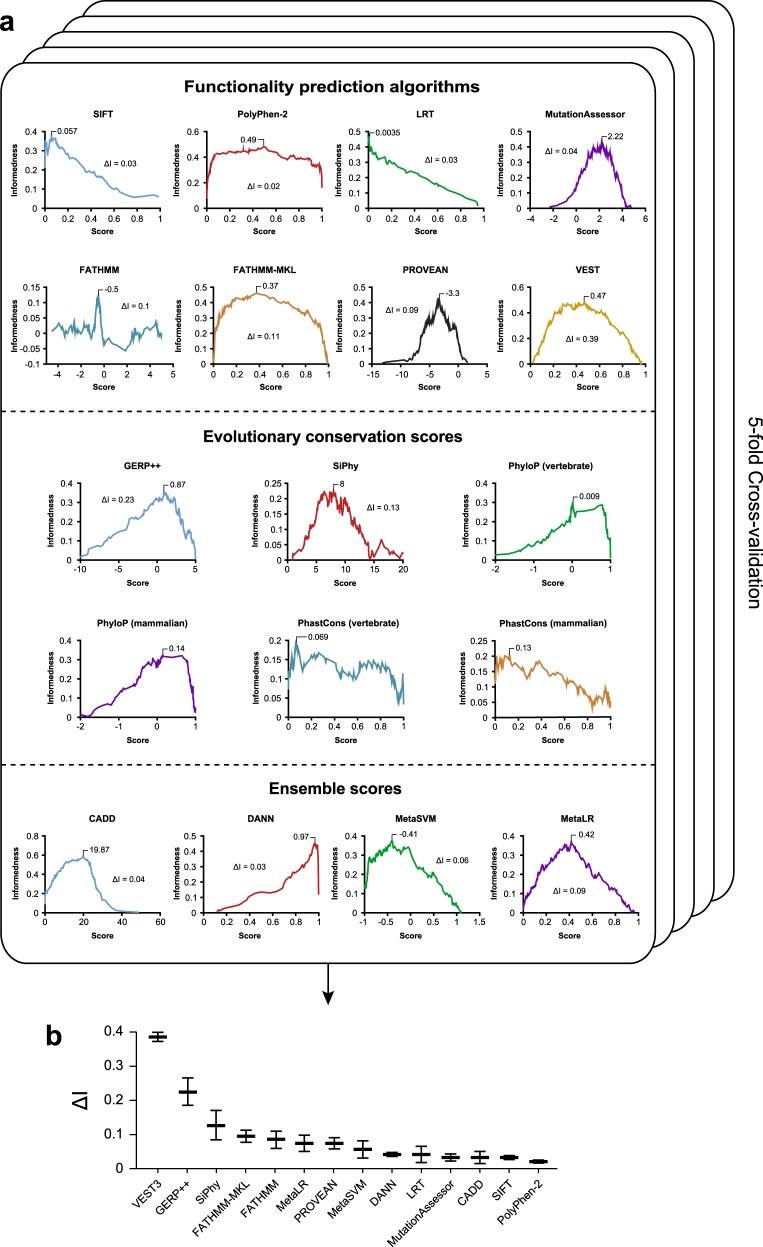
Pharmacogenetic threshold optimization results in substantially higher probabilities to make informed decisions. **a** The degree of informedness is plotted as a function of threshold score for eight functionality prediction algorithms, six evolutionalry conservation scores and four ensemble scores. The threshold score corresponding to the global maximum of informedness is indicated. ΔI denotes the gain in informedness between using the pharmacogenetically optimized threshold and the conventional threshold provided in the literature. Results are depicted for one of the five folds in our cross-validation analysis. **b** Averaging the ΔI values of the five folds demonstrates that the increases in informedness due to ADME-specific parameterization differ substantially between algorithms and are stable across folds. As no standard thresholds for PhyloP and PhastCons are provided in the literature, no ΔI values for these conservation scores are shown. Error bars indicate S.D.

We decided to optimize parameterization of the algorithms based on the concept of overall informedness, defined as the probability that a prediction is informed (i.e. not by chance) using the Youden index as statistical target metric (see Supplementary Figure 1 for graphical depiction and further explanation). The Youden index *J* developed as a measure to rate diagnostic tests [36], is defined on the basis of a ROC curve as $J = max_x\left\{ {sens\left( x \right) + spec\left( x \right) - 1} \right\}$ across all potential threshold scores *x*. The point x for which the sum of sensitivity and specificity is maximal indicates the optimal threshold value that maximizes the capacity of the test to differentiate between deleterious and neutral variants when sensitivity and specificity are weighted equally, thus avoiding impacts of the unequal distribution of neutral and functionally deleterious variants in our data set [37]. We defined the optimal threshold value for each algorithm or score based on the global maximum of the informedness graph (Fig. 3a). Interestingly, shapes of the informedness functions differed substantially between algorithms. While some algorithms, such as PolyPhen-2 and FATHMM-MKL showed largely stable informedness values across a wide range of threshold scores, others, such as SIFT or PROVEAN, exhibited sharp peaks, indicating drastic differences in the robustness of the method to variation in threshold scores.

To evaluate the sensitivity of this approach to variability in training set variants we performed 5-fold cross validations in which we partitioned the variants into five equally sized subsets. Of these five subsets, four are used for model training and one is used for independent validation. This process is iterated five times with each of the five subsamples serving once as validation data set. For most algorithms, including PROVEAN (|coefficient of variation| = 0.006), DANN (|CV| = 0.012) and VEST3 (|CV| = 0.054), the optimal threshold differed only marginally between folds, demonstrating the robustness of the threshold optimization (Supplementary Table 4). In contrast, optimal thresholds were substantially different across folds for PhyloP (|CV| = 3.12) and FATHMM (|CV|=2.33). Interestingly, the added value of threshold optimization differed substantially across prediction tools (Fig. 3b and Table 2). While threshold optimization did only marginally improve the informedness of PolyPhen-2, SIFT or CADD (ΔI < 0.03), the performance of other algorithms, such as SiPhy (ΔI = 0.13), GERP++(ΔI = 0.22) and VEST3 (ΔI = 0.38), were highly improved.

**Table 2:** Overview of computational method parameters to assess the functionality of pharmacogenetic variants

		Conventional			ADME optimized		
Algorithm	Category	Threshold	Sensitivity (%)	Specificity (%)	Threshold	Sensitivity (%)	Specificity (%)
SIFT	Functionality prediction algorithms	<0.05	80.7	54.2	<0.0376	75.6	57.6
PolyPhen-2		>0.447	80.8	63	>0.3841	83	61.6
LRT		<0.001	66.3	72.3	<0.0025	77.3	65.2
MutationAssessor		>1.9	79	63.7	>2.0566	74	67.8
FATHMM		<−1.5	18.2	81.9	<0.486	69.9	27.1
FATHMM-MKL		>0.73	64.2	68	>0.3982	77.4	63.3
PROVEAN		<−2.5	80.7	56.9	<−3.286	72.2	72.2
VEST3		>0.9	14.3	95.9	>0.4534	67.6	78.8
GERP++	Evolutionary conservation scores	>4.4	28.4	84.4	>1.2482	84.2	47.6
SiPhy		>12.17	32.1	78.2	>7.2442	51.9	72.7
PhyloP (vertebrate)		NA	NA	NA	>0.5216	70.5	53.7
PhyloP (mammalian)		NA	NA	NA	>0.0461	77.4	49
PhastCons (vertebrate)		NA	NA	NA	>0.07	81.1	34.7
PhastCons (mammalian)		NA	NA	NA	>0.1872	67.4	49.7
CADD	Ensemble scores	>15	75.8	74.8	>19.19	74.2	78.9
DANN		>0.99	68.9	70.1	>0.9688	85.8	54.4
MetaSVM		>0	43.4	86.3	>−0.3371	51.6	78.1
MetaLR		>0.5	41.2	84.2	>0.4039	52.2	76.7

Sensitivity and specificity of each prediction method is shown for conventional disease dataset-based parameterization and ADME optimized parameters. Threshold values are in arbitrary units, values for sensitivity and specificity are provided in percentage (%)

When integrating the individual predictions for each variant into a consensus decision by averaging the ADME-optimized thresholds across folds, the resulting model achieved 82% sensitivity and 62% specificity. To improve this predictive accuracy, we evaluated the predictive performance for all possible combinations of threshold-optimized algorithms. Importantly, optimal model constituents were highly similar between folds (Supplementary Table 4) and, based on these findings, we integrated the LRT, MutationAssessor, PROVEAN, VEST3 and CADD using ADME-optimized parameters (Table 2) into our pharmacogenetic prediction framework.

### Performance of ADME optimized prediction framework

**Fig. 4:**
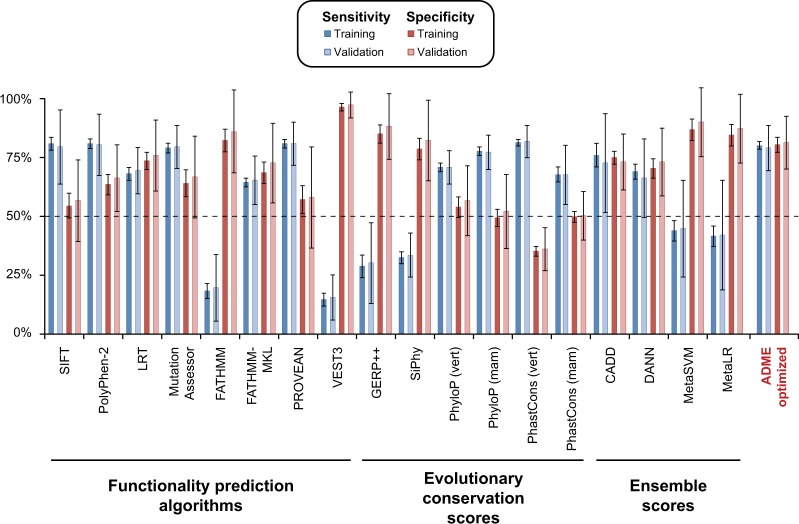
The ADME optimized model outperforms conventional methods for the functionality prediction of pharmacogenetic variants. Column plot showing the sensitivity (shades of blue) and specificity (shades of red) of commonly used functionality prediction algorithms, ensemble scores and evolutionary conversation scores as well as of the ADME optimized prediction model presented here. Notably, the ADME optimized model was the only method achieving both sensitivity and specificity of >80% in both training and validation data set. Error bars indicate S.D. across folds

In the training data sets the ADME optimized prediction framework achieved overall sensitivity and specificity of 80 ± 2% S.D. and 80 ± 3% S.D., respectively, thus outperforming all previously reported functionality prediction algorithms, conservation or ensemble scores. This superior performance of the ADME optimized model was validated using the independent variants from each training set, achieving sensitivity and specificity of 79 ± 10% S.D. and 81 ± 11% S.D., respectively (Fig. 4).

**Fig. 5:**
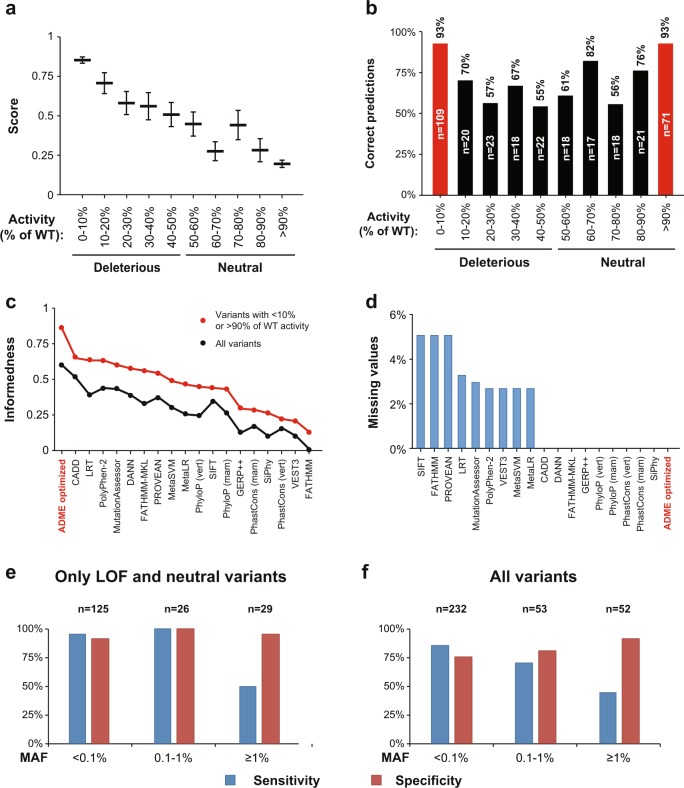
The ADME optimized model provides quantitative estimates of functional variant effects. **a** The score provided by the ADME optimized prediction model correlates quantitatively with the level of gene product functionality determined experimentally in vitro (*R*^2^ = 0.9, *p* = 2.9 × 10^−5^). Highest scores are provided for LOF variants with <10% of WT functionality (0.84 ± 0.02 s.e.m.), while variants that do not affect gene product functionality receive lowest scores (0.19 ± 0.02 s.e.m.). Data is plotted as mean ± s.e.m. **b** 93% of variants that resulted in severely decreased functionality in vitro (<10% activity of WT) were correctly classified as deleterious, whereas variants whose effect on functionality was only moderate (decreased functionality variants; 10–50% activity of WT), were flagged with lower probabilities. Similarly, variants that showed equivalent activity than WT (>90%) were more likely to be flagged as functionally neutral (93% specificity) than variants with 50–90% of activity. **c** Levels of informedness are shown for all variants (black) and variants with <10% and >90% of WT activity (red curves corresponding to red columns in **b**). Note that the ADME optimized prediction framework achieved the highest values of informedness, irrespective of which variants were considered. **d** Overview of the fraction of variants for which no prediction could be obtained by the individual algorithms. While SIFT, FATHMM and PROVEAN did not return predictions for 5% of variants, CADD, DANN, SiPhy, PhastCons, PhyloP, GERP and the ADME optimized model provided assessments for all non-synonymous variants analyzed here. **e**, **f** Column plot depicting sensitivity and specificity of the ADME optimized prediction model for LOF and functionally neutral variants (**e**) or all variants (**f**) depending on their minor allele frequencies (MAF). Note that predictive measures are higher for very rare (MAF < 0.1%) and rare variants (0.1% ≤ MAF < 1%) compared to common variants (MAF ≥ 1%). vert vertebrate, mam mammalian

Importantly, when analyzing all 337 pharmacogenetic variants using the developed ADME-optimized prediction framework, we found that the score of the ADME-optimized prediction model correlates well with the extent of functional impact of the variant in question (*R*^2^ = 0.9, *p* = 2.9 × 10^−5^; Fig. 5a). For LOF variants (<10% activity of WT) the model yielded scores of 0.84 ± 0.02 s.e.m., which continuously decreased with increased functionality in vitro up to 0.19 ± 0.02 s.e.m. for functionally neutral variants (>90% activity of WT). When translating these scores into dichotomous functionality predictions, the model achieved 93% sensitivity (101/109 variants) for LOF variants that decreased activity >10-fold whereas variants with only mild functional effects were recognized with 55–70% sensitivity (Fig. 5b). Conversely, prediction specificity for variants that exhibited >90% of the functional activity of the WT allele, was 93% (66/71 variants), whereas the specificity for variants with 50–100% activity was only 56–82% . Overall, these performance metrics resulted in a predictive accuracy of 93% for LOF and functionally neutral variants, compared to 84% for CADD, the score with the next highest accuracy.

Overall, the ADME optimized model achieved the highest extent of informedness for LOF and neutral variants (*I*_ADME_ = 0.86), followed by CADD (*I*_CADD_ = 0.65) and LRT (*I*_LRT_ = 0.63; Fig. 5c). Similarly, when all variants are considered and classified dichotomously, the ADME model substantially outperformed current models (*I*_ADME_ = 0.6 followed by *I*_CADD_ = 0.51). In contrast, VEST3 and FATHMM only yielded overall values of *I*_VEST_ = 0.11 and *I*_FATHMM_ = 0.01, respectively. Besides the increased predictive power, the integrated ADME model successfully derived assessments for all variants, while some individual algorithms were unable to predict the functional impact of up to 5% of all variants analyzed (Fig. 5d).

Lastly, we analyzed whether the predictive performance of the ADME optimized prediction model depended on the frequency of the respective variant. The majority of the 337 variants analyzed in this study were rare (*n* = 285) or very rare (*n* = 232) with MAF < 1% or MAF < 0.1%, respectively. Notably, the predictive power of the model for LOF and functionally neutral variants was better for very rare (*I*_MAF < 1%_ = 0.87) and rare mutations (*I*_0.1%≤ MAF <1%_ = 1) compared to common variants (*I*_MAF ≥ 1%_ = 0.45; Fig. 5e). Similar trends were observed when all variants were considered either in our model (Fig. 5f) or in individually tested algorithms (Supplementary Figure 2). While our results correlated significantly with data from REVEL (*R*^2^ = 0.5; Supplementary Figure 3), a prediction method to analyze the pathogenicity of rare missense variants [38], the ADME optimized prediction framework performed substantially better for the prediction of pharmacogenetic variants: When using the threshold score that resulted in the best Youden index for disease associated variants (0.5), REVEL achieved informedness values of 0.36 and 0.61 on our pharmacogenetic data set when considering all or only LOF and functionally neutral variants, respectively. In contrast, on the same variants the ADME-optimized model achieved informedness levels of 0.6 and 0.86, respectively (Supplementary Table 5). These findings emphasize the usefulness of the ADME optimized prediction model for the functional interpretation of pharmacogenetic variants with low frequencies, which, due to their large numbers, are difficult to systematically characterize in vitro.

## Discussion

Despite the abundance of genomic data generated in the frame of multiple completed and ongoing population-scale sequencing projects, the understanding of personal genomic data and translation into clinically actionable information is still very limited. Functional interpretation of identified mutations relies either on clinical or experimental data, which is only available for a small subset of well-characterized genetic variants, or on computational prediction tools. A vast number of algorithms and scores have been presented that predict the likelihood of whether a genetic variant has a functional impact based on sequence homology, structural features, preexisting annotations or, most importantly, evolutionary constraints [39] and these tools have been reasonably successful in predicting mutations associated with disease [40, 41]. However, the predictive quality of these algorithms on specific classes of genes with lower evolutionary constraints that are often not directly disease-associated, has not been evaluated.

Here, we benchmarked 18 commonly used prediction methods on a pharmacogenetic data set encompassing 337 variants with available high-quality experimental characterization data using functional assays, which have been suggested as gold standard sets for the benchmarking of computational tools [42]. We focused on pharmacokinetic genes involved in drug metabolism and transport as these can be genetically highly polymorphic and are subject to low evolutionary constraints. In contrast, drug targets are highly heterogeneous regarding their evolutionary conservation and are commonly associated with congenital diseases [43]. Importantly, we found that performance of tested algorithms on this ADME data set was substantially lower than on data sets comprising of pathogenic variants (Table 1). Of the different methods tested, evolutionary conservation scores exhibited overall the worst performance, supporting our hypothesis that selective constraints are unreliable measures for assessing the functionality of variants in genes with low evolutionary pressure, such as ADME genes [44]. Given that most algorithms rely on evolutionary conservation as a core feature, these findings suggest that ADME gene-specific parameter optimization and integration of orthogonal approaches represent an appealing rationale to improve the pharmacogenetic predictions.

After optimization our model significantly outperformed all individual functionality prediction methods achieving a predictive accuracy of 93% for LOF and functionally neutral variants, compared to 84% for CADD, the second best algorithm. Interestingly, we achieved the best overall performance not by integrating the individually best performing algorithms. For instance, LRT ranked only as 5 with an accuracy of 81.8% but the LRT score was integrated into the most predictive ADME model. This finding is in agreement with the performance of the model on human disease alleles for which the overlap between LRT and other methods has been shown to be low [23].

Deviations between in vitro data and in silico predictions can be allotted to both computational and experimental factors [45]. Firstly, the use of sensitivity and specificity as statistical summary metrics requires dichotomous variant classification, which relies on the definition of an activity threshold below which variants are considered as deleterious (here 50% of WT) and modulation of this cutoff will influence the number of discrepancies. On our pharmacogenetic data set the sensitivity and specificity of predictions was substantially higher for variants that caused >10-fold reduction or no reduction (activity ≥ 90%) in protein functionality, respectively, compared to variants that only had moderate effects (Fig. 5b), indicating that the choice of a more stringent threshold would further improve predictive performance.

Secondly, inter-experimental variability can change the classification of a variant, particularly for variants that result only in moderate decreases of protein activity; a problem which can only be overcome by stringent experimental replications. Furthermore, variants that result in substrate-specific functionality changes can be missed when probing functionality using a limited number of assays (Supplementary Table 2). We observed substrate-dependent differences for CYP2D6*49, which significantly reduces enzyme activity towards the CYP2D6 substrates dextromethorphan and bufuralol but does not affect the clearance of tamoxifen [46, 47]. Similarly, CYP2C8*10 and CYP2C8*13 exhibited reduced amodiaquine *N*-deethylation activity while their paclitaxel hydroxylation kinetics remained unaffected [48].

Lastly, discrepancies can occur between the functional impact of a variant in vitro and in vivo. One such example is CYP2D6*35, which shows reduced tamoxifen hydroxylation in vitro [47] but has not been associated with reduced activity in vivo [49]. Similarly, CYP2A6*8 is unlikely to affect catalytic activity in vivo [50] but strongly impairs nicotine and coumarin metabolism in vitro [51]. Our ADME optimized prediction model clearly flagged both alleles as functionally neutral (Fig. 2a), thus correctly predicting the functional consequence in vivo. However, for the sake of consistency and clarity we trained our model exclusively with quantitative and homogeneous experimental in vitro data and did not introduce more heterogeneous and variable data from patient phenotyping.

The presented prediction framework improved both sensitivity and specificity of functionality predictions for variants in poorly conserved genes compared to preexisting assays. However, while the model is capable of predicting the functionality of genetic variations beyond missense mutations, such as indels, frameshifts and synonymous variants, comprehensive investigations into the performance regarding these variant classes are currently not feasible due to the small number of such pharmacogenetic variants with available experimental characterization data.

In summary, we have developed and validated a functionality prediction framework for genetic variants in ADME genes that significantly outperforms current methods using multiple quality metrics, is not limited to previously encountered mutations and can be easily applied to novel variants through the use of the established ANNOVAR platform. Importantly, the model not only informs about the likelihood that the variant in question has deleterious effects on the functionality of the gene product but also provides quantitative estimates of its effect on gene function. Thus, it presents a versatile tool that aspires to improve the prediction of phenotypic consequences of variants discovered in genomic sequencing projects, thereby facilitating the translation of the entire spectrum of patient’s genetic variability into pharmacogenetic recommendations.

## Electronic supplementary material

Supplementary Figure 1

Supplementary Figure 2

Supplementary Figure 3

Supplementary Table 1

Supplementary Table 2

Supplementary Table 3

Supplementary Table 4

Supplementary Table 5

Track changes MS
