# Supplementary material for: An optimized prediction framework to assess the functional impact of pharmacogenetic variants
Source: Pharmacogenomics J. 2018 Sep 12;19(2):115–26. doi: 10.1038/s41397-018-0044-2 (PMC6462826; doi:10.1038/s41397-018-0044-2)
Supplement: Supplementary file 1 — Supplementary Figure 1 [file 41397_2018_44_MOESM1_ESM.pdf]

Supplementary Figure 1

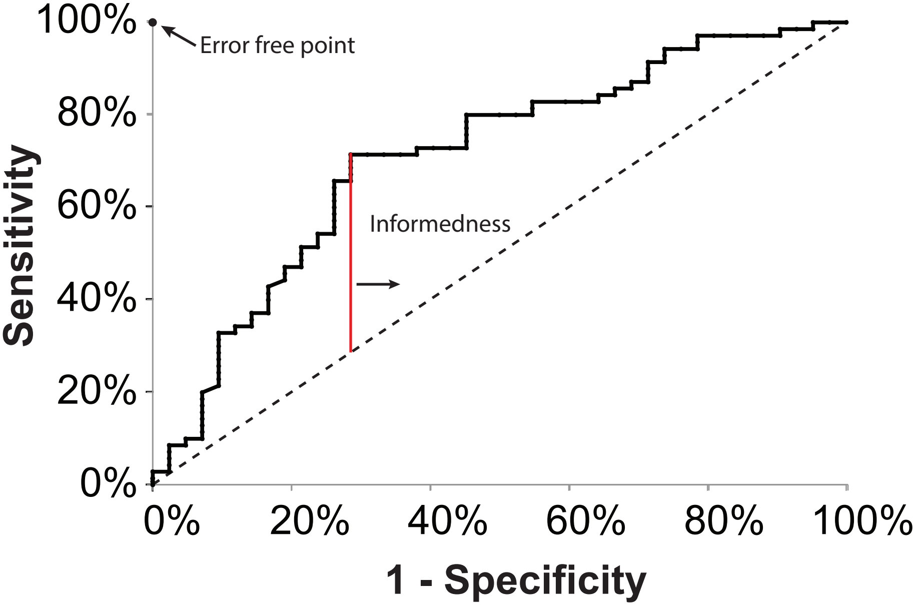

Supplementary Figure 1: Schematic depiction of the Youden Index and its derivation from an exemplary receiver operating characteristic (ROC) curve. The Youden index (red line) is the vertical distance between the ROC curve (black line) and the bisectrix (dashed line). Values on the bisectrix (i.e. Youden index = 0) correspond to the inability to discriminate between functional and neutral variants and deciding on the functionality of variants solely by chance. A Youden index of 1 indicates perfect informedness, i.e. 100% sensitivity and 100% specificity. The point at which the Youden index is maximal and closest to the error-free point (0,1) corresponds to the highest probability to make an informed decision and is selected as the ideal threshold score to separate deleterious and function-
