# Supplementary material for: An optimized prediction framework to assess the functional impact of pharmacogenetic variants
Source: Pharmacogenomics J. 2018 Sep 12;19(2):115–26. doi: 10.1038/s41397-018-0044-2 (PMC6462826; doi:10.1038/s41397-018-0044-2)
Supplement: Supplementary file 2 — Supplementary Figure 2 [file 41397_2018_44_MOESM2_ESM.pdf]

Supplementary Figure 2

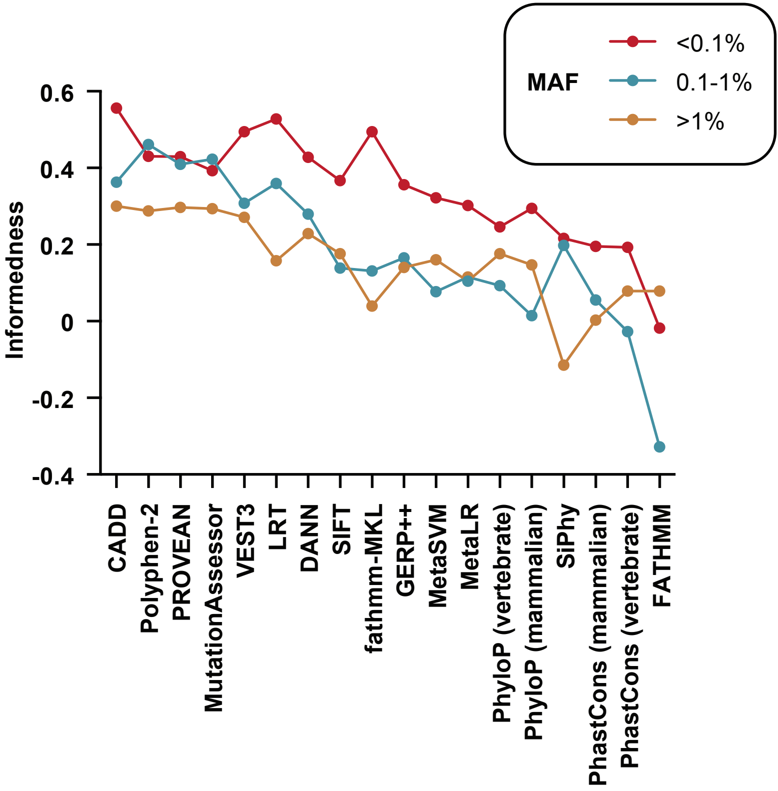

Supplementary Figure 2: Functionality prediction algorithms and scores perform overall better on rare compared to common pharmacogenetic variants. Informedness values are shown for variants binned by their minor allele frequency (MAF):<0.1% (n=232, red), 0.1-1% (n=53, blue) and >1% (n=52, yellow)
