# Supplementary material for: An optimized prediction framework to assess the functional impact of pharmacogenetic variants
Source: Pharmacogenomics J. 2018 Sep 12;19(2):115–26. doi: 10.1038/s41397-018-0044-2 (PMC6462826; doi:10.1038/s41397-018-0044-2)
Supplement: Supplementary file 3 — Supplementary Figure 3 [file 41397_2018_44_MOESM3_ESM.pdf]

Supplementary Figure 3

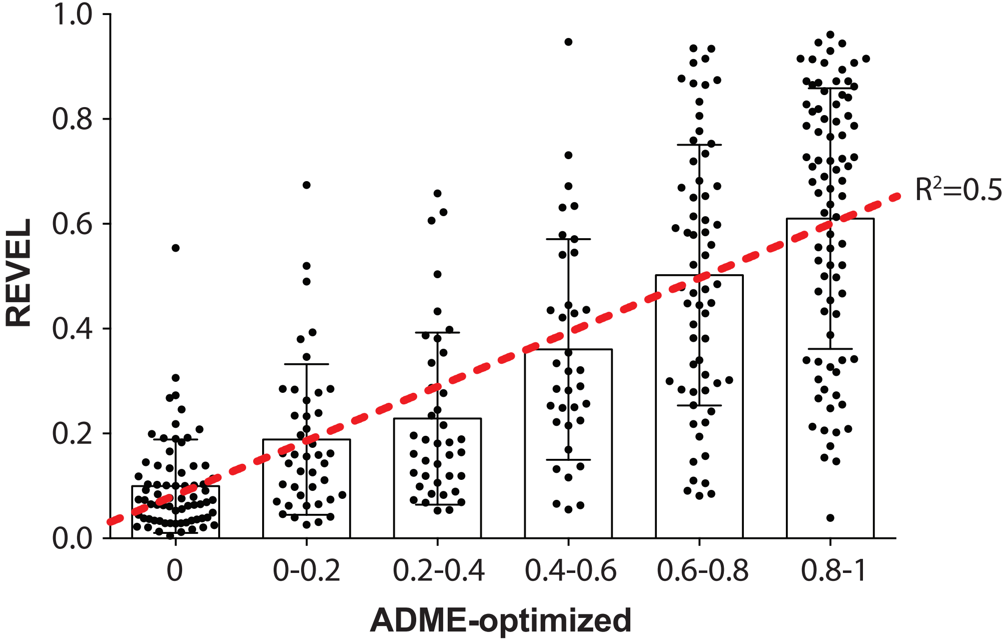

Supplementary Figure 3: Scores derived by the ADME-optimized prediction model correlate with REVEL scores. Note that while the scores between the models overall correlate, predictions for individual variants can diverge substantially. Dashed red line indicated linear regression.
